# Cell-Penetrating Peptides-Based Liposomal Delivery System Enhanced Immunogenicity of Peptide-Based Vaccine against Group A Streptococcus

**DOI:** 10.3390/vaccines9050499

**Published:** 2021-05-12

**Authors:** Jieru Yang, Farrhana Firdaus, Armira Azuar, Zeinab G. Khalil, Nirmal Marasini, Robert J. Capon, Waleed M. Hussein, Istvan Toth, Mariusz Skwarczynski

**Affiliations:** 1 School of Chemistry and Molecular Biosciences, The University of Queensland, St. Lucia, QLD 4072, Australia; j.yang@uq.net.au (J.Y.); farrhana.firdaus@uq.net.au (F.F.); armira.azuar@uq.net.au (A.A.); w.hussein@uq.edu.au (W.M.H.); i.toth@uq.edu.au (I.T.); 2 Institute for Molecular Bioscience, The University of Queensland, St. Lucia, QLD 4072, Australia; z.khalil@uq.edu.au (Z.G.K.); r.capon@imb.uq.edu.au (R.J.C.); 3 School of Biomedical Sciences, The University of Queensland, St. Lucia, QLD 4072, Australia; n.marasini@uq.edu.au; 4 School of Pharmacy, The University of Queensland, Woolloongabba, QLD 4102, Australia

**Keywords:** cell-penetrating peptide, vaccine delivery, peptide-based vaccine, liposomes, group A streptococcus

## Abstract

Peptide-based vaccine development represents a highly promising strategy for preventing Group A Streptococcus (GAS) infection. However, these vaccines need to be administered with the help of a delivery system and/or immune adjuvant. Cell-penetrating peptides (CPPs) have been used as a powerful tool for delivering various therapeutic agents, including peptides, as they can overcome the permeability barrier of cell membranes. Here, we used CPPs to deliver our lead lipopeptide-based vaccine (LCP-1). CPPs were anchored through a spacer to LCP-1-bearing multilamellar and unilamellar liposomes and administered to Swiss outbred mice. Tat_47–57_ conjugated to two palmitic acids via a (Gly)_6_ spacer (to form a liposome-anchoring moiety) was the most efficient system for triggering immune responses when combined with multilamellar liposomes bearing LCP-1. The immunostimulatory potential of a variety of other CPPs was examined following intranasal administration in mice. Among them, LCP-1/liposomes/Tat_47–57_ and LCP-1/liposomes/KALA induced the highest antibody titers. The antibodies produced showed high opsonic activity against clinically isolated GAS strains D3840 and GC2 203. The use of the CPP-liposome delivery system is a promising strategy for liposome-based GAS vaccine development.

## 1. Introduction

Peptide-based subunit vaccines exhibit an improved safety profile in comparison to conventional vaccines because they utilize only small antigens derived from a target pathogen. As such, they are free from redundant pathogen components, which reduce the possibility of inducing allergic or autoimmune responses. Moreover, peptide-based vaccines use synthetic peptides as antigens; these can be produced relatively easily and cost-effectively without any risk of biological contamination. However, peptide antigens are poorly immunogenic. Therefore, immune stimulators, namely adjuvants, are required in peptide-based vaccine formulations to improve efficacy.

Unfortunately, the rate of discovery in terms of effective and safe adjuvants is slower than that of discovering antigens. Currently, only a limited number of adjuvants are licensed for human vaccines, and those are not always effective when used with specific antigenic peptides [1,2]. Producing a suitable adjuvant remains an undeniable challenge in the development of peptide-based vaccines. Most adjuvants are designed to target pattern recognition receptors (PRRs), particularly toll-like receptors (TLRs), expressed on antigen-presenting cells (APCs) to achieve enhanced uptake of co-delivered antigen. However, other strategies to enhance antigen uptake by APCs have also been examined [3,4,5]. CPPs are a group of short peptides that have the special ability to overcome the permeability barrier of cell membranes and enter the cell interior in a non-invasive manner without assistance from membrane proteins [6]. Thus, they have been thoroughly explored for delivering various cargos, such as peptides, nucleic acids, proteins, nanoparticles, and liposomes, into cells [7,8,9]. CPPs have also been investigated in the vaccine delivery field over the past decade to facilitate antigen delivery into cells [10]. Although the internalization mechanisms of CPPs remain unclear, CPPs have already been employed to enhance both cellular immune responses via the delivery of antigen directly into the cytoplasm, and humoral immune responses, where antigens are delivered through the endocytic pathway [11,12,13].

Group A Streptococcus (GAS) is a Gram-positive human pathogen responsible for a plethora of diseases, ranging from non-invasive (e.g., pharyngitis and impetigo) to post-infectious diseases (e.g., rheumatic fever (RF) and rheumatic heart disease (RHD)). Pharyngitis is the most common GAS infectious disease, with over 600 million people treated worldwide, annually [14]. It is estimated that up to 3% of pharyngitis patients eventually develop acute RF which can evolve into RHD [15,16,17]. In 2015, over 30 million cases of rheumatic heart disease were observed globally [15]. Both RF and RHD are autoimmune diseases trigger by untreated or frequent GAS infections. These autoimmune responses are most likely generated due to the similar structure of human and GAS proteins, which activate antibodies and/or T cell responses against human proteins [18]. There are no licensed GAS vaccines currently available on the market despite nearly a century of work in this space. The earliest attempts to develop a GAS vaccine can be tracked back to the early 20th century, when live-attenuated or inactivated whole organisms were investigated [19,20]. However, these vaccine candidates stimulated allergic and autoimmune responses and provide protection against a narrow range of GAS strains [21,22]. As GAS infection is associated with an M-protein activity, further vaccine development focused mostly on this protein. However, the strategy of utilizing the whole M protein as an antigen has been abandoned due to the risk of autoimmune response [23], which triggered RF among the vaccinated children in the clinical trial [24]. Besides, a large number of studies reported that the M protein comprises the cross-reactive B and T cell epitopes with human tissues [25,26,27], and the immunization with M protein may not produce robust immune responses against a variety of GAS serotypes. Consequently, non-cross-reactive peptide fragments of the M-protein have been used instead as a suitable alternative for GAS vaccine development [28]. For example, an M-protein-derived B-cell epitope (J8 peptide: QAEDKVKQSREAKKQVEKALKQLEDKVQ) has been used in peptide-based GAS vaccine design [29] and recently reached phase I clinical trial [30]. Furthermore, lipid-core-peptide (LCP) systems have been frequently utilized in GAS vaccine studies [31,32,33]. LCP is a self-adjuvanting carrier system consisting of lipoamino acids (LAAs), a branching moiety and conjugated peptide. LAAs can be recognized by toll-like receptor-2 (TLR-2) on dendritic cells, thus they act as self-adjuvanting moieties, and conjugation between lipids and peptides can prevent enzymatic degradation of the peptides [33].

**Figure 1:**
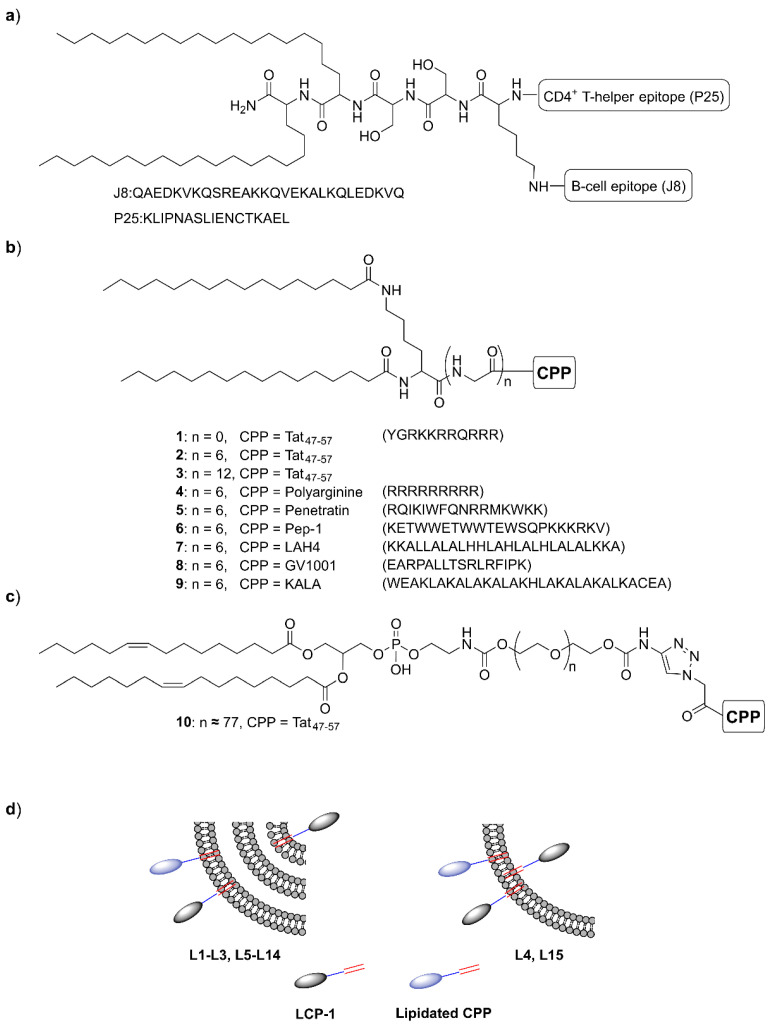
The CPP-liposome-based vaccine delivery system. (**a**) The chemical structure of LCP-1; (**b**) the chemical structure of **1–9** (lipidated CPPs); (**c**) the chemical structure of **10** (DOPE-PEG_3400_-Tat_47–57_); (**d**) schematic representation of the multilamellar liposomes **L1–L3, L5–L14** and unilamellar liposomes **L4** and **L15** (Table 1).

Here, we produced a lipopeptide GAS vaccine (LCP-1) consisting of B-cell epitope (J8), universal T-helper epitope (P25), and a branching lysine and lipopeptide adjuvating moiety (C20 lipoamino acid, 2-amino-d,l-eicosanoic acid) (Figure 1a), which was further entrapped in liposomes. CPPs were then incorporated into these liposomes to enhance vaccine efficacy (Figure 1). To allow for CPP co-delivery with liposomes, a lipidic tail was conjugated to the *N*-terminal of the CPPs as a liposome anchoring moiety. Tat_47–57_ was selected as a model CPP and the optimal CPP-liposome anchoring strategy was identified. The immunostimulatory potential of a variety of CPPs was then examined. All LCP-1/liposomes/CPP systems were evaluated in mice following intranasal administration.

## 2. Materials and Methods

### 2.1. Materials

Analytical-grade, or equivalent, chemicals were used in this study unless stated otherwise. Diethyl acetamidomalonate (DAAM), 1-bromo-octadecane, sodium ethoxide, 5,5-dimethyl-1,3-cyclohexanodione (Dimedone), acetic acid, 4-dimethylaminopyridine (DMAP), dicyclohexylcarbodiimide (DCC), triethylamine (TEA), ethanol, and protected Fmoc/Boc-amino acids were obtained from Reanal (Budapest, Hungary) or Novabiochem (Läufelfingen, Switzerland). 1-[Bis(dimethylamino)methylene]-1*H*-1,2,3-triazolo [4,5-b]pyridinium 3-oxid hexafluorophosphate (HATU) was purchased from Mimotopes (Melbourne, Australia). *p*-MBHA‧HCl resin was purchased from Peptides International (Louisville, KY, USA). Rink amide 4-methylbenzhydrylamine (MBHA) resin was purchased from Novabiochem (Hohenbrunn, Germany). Methanol, dichloromethane (DCM), *N,N’*-dimethylformamide (DMF), chloroform, *N,N*-diisopropylethylamine (DIPEA), HPLC grade acetonitrile, trifluoroacetic acid (TFA), and piperidine were purchased from Merck (Hohenbrunn, Germany). Triisopropylsilane (TIPS), copper wire, phenylmethylsulfonyl fluoride (PMSF), and cholera toxin B subunit (CTB) were purchased from Thermo Scientific (Victoria, Australia). Phosphate-buffered saline (PBS) tablets were purchased from Gibco (Paisly, UK). *O*-phenylenediamine dihydrochloride (OPD) was purchased from SIGMAFAST^TM^. Goat anti-mouse IgG conjugated to horseradish peroxidase and its substrate were purchased from Bio-Rad (California, CA, USA). Horseradish peroxidase-conjugated anti-mouse IgG1 and IgG2a were purchased from Sigma-Aldrich (Castle Hill, Australia). Dipalmitoylphosphatidylcholine (DPPC), cholesterol (CH), an Avanti Mini Extruder, PC membranes, and filter supports were purchased from Avanti (Alabaster, AL). Iscove’s Modified Dulbecco’s Medium (IMDM), fetal bovine serum (FBS), Penicillin-Streptomycin, Dulbecco’s Modified Eagle Medium (DMEM), and IC fixation buffer, snakeskin pleated dialysis tube, 3-(4,5-dimethylthiazol-2-yl)-2,5-diphenyltetrazolium bromide (MTT), RPMI 1640 medium, horse blood, and Todd-Hewitt Broth were purchased from Thermo Fisher Scientific (Life Technologies, Scoresby, VIC, Australia).

Analytical reverse-phase high-performance liquid chromatography (RP-HPLC) was performed on a Shimadzu LCMS-2020 instrument (Kyoto, Japan) with a Vydac analytical C-4 (214TP; 10 µm, 250 × 4.6 mm) or C-18 column (218TP; 10 µm, 250 × 4.6 mm) and a flow rate of 1 mL/min. Detection was at 214 nm. Preparative RP-HPLC was performed on a Shimadzu instrument using either a Vydac or an Altima preparative C-18 column (218TP; 10 mm, 250 × 22 mm) and a C-4 column (214TP; 10 mm, 250 × 22 mm) or a semi-preparative column in linear gradient mode using a flow rate of 10–20 mL/min. Detection was conducted at 214 nm.

### 2.2. Methods

#### 2.2.1. LCP-1 Synthesis

LCP-1 was synthesized at a 0.2 mmol scale using *tert*-butyloxycarbonyl (Boc) solid-phase peptide synthesis (SPPS). 4-Methylbenzhydrylamine (MBHA, substitution ratio: 0.59 mmol/g, 0.20 mmol, 0.34 g) resin was swelled in *N,N’*-dimethylformamide (DMF) overnight. Dde-C20-OH (0.84 mmol, 0.41 g, 4.0 equiv.) was activated by 0.5 M HATU (0.80 mmol, 1.6 mL, 4.0 equiv.) and DIPEA (1.2 mmol, 0.22 mL, 6.2 equiv.), then coupled to resin for 2 × 1 h periods at room temperature (RT). Deprotection of Dde was performed with 5% hydrazine monohydrate/DMF at RT for 2 × 10 min and 1 × 20 min periods. The subsequent amino acids (0.84 mmol, 4.0 equiv.) were activated by DIPEA (1.2 mmol, 0.22 mL, 6.2 equiv.) and 0.5 M HATU (0.80 mmol, 1.6 mL, 4.0 equiv.) and coupled twice at 70 °C for 5 min and 10 min, respectively, via a CEM Discover reactor on SPS mode. Boc was deprotected by adding neat TFA for 2 × 1 min periods at RT. Peptide cleavage from the resin was performed using anhydrous hydrogen fluoride (10 mL hydrofluoric acid/g resin) and scavenger (5% (*v/v*) p-cresol and 5% (*v/v*) p-thiocresol) solution at −8 °C [34]. The crude peptides were washed with cold diethyl ether, dissolved with acetonitrile:water (1:1) and lyophilized. The crude LCP-1 was purified using RP-HPLC on a C-4 Vydac column with a 40%–70% solvent B gradient over 30 min at a flow rate of 20 mL/min (Solvent B: 90% MeCN/0.1% TFA/H_2_O; Solvent A: 0.1% TFA/H_2_O). The pure compound was analyzed using analytical HPLC and electrospray ionization mass spectrometry (ESI-MS). HPLC analysis (C-4 column): *t*_R_ = 36.5 min, purity: >95%. Yield: 9%. ESI-MS: m/z 1021.9 (calc: 1022.1) [M+6H]^6+^; 876.5 (calc: 876.2) [M+6H]^7+^; 767.5 (calc: 766.9) [M+8H]^8+^; 681.6 (calc: 681.7) [M+9H]^9+^. MW: 6126.6 Da (see Supporting Information, Figure S1).

#### 2.2.2. Synthesis of Cell-Penetrating Peptides and their Conjugates

Tat_47–57_ and CPP conjugates (compounds **1–9**, Figure 1) were synthesized in a similar manner to LCP-1, using standard Boc-SPPS, as described above. Two palmitic acid moieties were coupled to the *N*-terminus lysine for compounds **1–9**.

Tat_47–57_ purity: >95%. Yield: 56%. ESI-MS: 780.2 (calc: 780.4) [M+2H]^2+^; 520.7 (calc: 520.6) [M+3H]^3+^. MW 1558.8 Da. *t*_R_ = 16.8 min (10–30% solvent B, 40 min, C18 column) (see Supporting Information, Figure S2).

Compound **1** purity: >95%. Yield: 26%. ESI-MS: m/z 722.2 (calc: 721.8) [M+3H]^3+^; 541.9 (calc: 541.6) [M+4H]^4+^; 433.9 (calc: 433.5) [M+ 5H]^5+^. MW 2162.5 Da. *t*_R_ = 33.0 min (30–60% solvent B, 40 min, C4 column) (see Supporting Information, Figure S3).

Compound **2** purity: >95%. Yield: 18%. ESI-MS: m/z 1254.4 (calc: 1254.1) [M+ 2H]^2+^; 836.3 (calc: 836.4) [M+ 3H]^3+^; 627.8 (calc: 627.6) [M+ 4H]^4+^. MW 2506.2 Da. *t*_R_ = 32.8 min (30–60% solvent B, 40 min, C4 column) (see Supporting Information, Figure S4).

Compound **3** purity: >95%. Yield: 19%. ESI-MS: m/z 713.0 (calc: 712.9) [M+4H]^4+^; 571.0 (calc: 570.5) [M+5H]^5+^. MW 2847.5 Da. *t*_R_ = 27.2 min (30–60% solvent B, 40 min, C4 column) (see Supporting Information, Figure S5).

Compound **4** purity: >95%. Yield: 18%. ESI-MS: m/z 791.0 (calc: 791.4) [M+ 3H]^3+^; 593.8 (calc: 593.8) [M+ 4H]^4+^. MW: 2371.1 Da. *t*_R =_ 23.07 min (25–55% solvent B, 40 min, C4 column) (see Supporting Information, Figure S6).

Compound **5** purity: >97%. Yield: 26%. ESI-MS: m/z 1066.0 (calc: 1065.7) [M+ 3H]^3+^; 799.0 (calc: 799.5) [M+4H]^4+^; 640.0 (calc: 639.8) [M+5H]^5+^. MW: 3194.0 Da. *t*_R_ = 24.12 min (40–70% solvent B, 40 min, C4 column) (see Supporting Information, Figure S7).

Compound **6** purity: >97%. Yield: 28%. ESI-MS: m/z 1266.0 (calc: 1265.7) [M+ 3H]^3+^; 949.7 (calc: 949.5) [M+ 4H]^4+^; 759.3 (calc: 759.8) [M+ 5H]^5+^. MW: 3974.0 Da. *t*_R_ = 24.9 min (35–65% solvent B, 40 min, C4 column) (see Supporting Information, Figure S8).

Compound **7** purity: >98%. Yield: 36%. ESI-MS: m/z 933.0 (calc: 932.2) [M+4H]^4+^; 746.0 (calc: 745.9) [M+5H]^5+^; 622.0 (calc: 621.8) [M+3H]^6+^; MW: 3724.8 Da. *t*_R_ = 28.6 min (40–65% solvent B, 40 min, C4 column) (see Supporting Information, Figure S9).

Compound **8** purity: >96%. Yield: 22%. ESI-MS: m/z 939.0 (calc: 939.3) [M+ 3H]^3+^; 705.0 (calc: 704.8) [M+ 4H]^4+^. MW: 2815.0 Da. *t*_R_ = 25.14 min (40–70% solvent B, 40 min, C4 column) (see Supporting Information, Figure S10).

Compound **9** purity: >98%. Yield: 28%. ESI-MS: m/z 1021.0 (calc: 1020.5) [M+4H]^4+^; 817.0 (calc: 816.6) [M+5H]^5+^; 681.0 (calc: 680.7) [M+3H]^6+^; 584.0 (calc: 583.6) [M+3H]^6+^; MW: 4078.2 Da. *t*_R_ = 28.2 min (40–70% solvent B, 40 min, C4 column) (see Supporting Information, Figure S11).

Compound **10** was synthesized based on a copper-catalyzed alkyne-azide 1,3-dipolar cycloaddition (CuAAC) ‘‘click” reaction to conjugate DOPE-PEG_3400_-alkyne to the azide-Tat_47–57_ (see Supporting Information, Figure S12)_._ Tat_47–57_ was synthesized using Boc-SPPS, then purified by RP-HPLC. The purified Tat_47–57_ was further modified with 2-azidoacetic acid to produce azide-Tat_47–57_. DOPE-PEG_3400_-alkyne was synthesized as detailed previously by Hussein et al. [35]. Then, a mixture of peptide azide-Tat_47–57_ (1.68 mg, 1.023 µmol, 2 equiv.) and DOPE-PEG_3400_-alkyne (2.2 mg, 0.511 µmol, 2 equiv.) was dissolved in DMF (1 mL) in a charged 2 mL round bottom flask. Copper wire (60–80 mg) was added and the oxygen partially removed from the flask via nitrogen bubbling for 30 s; the flask was then fully covered with aluminum foil. The reaction mixture was stirred at 50 °C under nitrogen atmosphere for 14 h. The wires were filtered off from the resulting product and washed with 1 mL of DMF. The DMF solution was slowly added to 4 mL of water (Rate: 0.005 mL/min). Compound **10** was formed via self-assembly and purified by dialysis over 3 days (Milli-Q water changed three times per day). After lyophilization, pure **10** was obtained as an amorphous white powder (0.56 mg, 27%). The quality of **10** was checked by matrix-assisted laser desorption/ionization (MALDI) (see Supporting Information, Figure S13).

#### 2.2.3. Preparation of LCP-1-Loaded Liposomes

LCP-1-loaded liposomes (**L1**–**L15**, Table 1) were produced by thin-film formation, followed by re-hydration with or without extrusion. The liposomes were formulated with DPPC, CH, LCP-1, and CPPs at a molar ratio of 2:1:0.05:0.01. DPPC (4 mg in 1 mL chloroform), CH (1.05 mg in 1 mL chloroform), CPPs (0.086 mg compound **1** in 0.5 mL methanol to formulate **L2**; 0.096 mg compound **2** in 0.5 mL methanol to formulate **L3** and **L4**; 0.48 mg compound **2** in 0.5 mL methanol to formulate **L5**; 0.183 mg compound **10** in 0.5 mL methanol to formulate **L6**; 0.105 mg compound **3** in 0.5 mL methanol to formulate **L8;** 0.095 mg compound **4** in 0.5 mL methanol to formulate **L9**; 0.1085 mg compound **5** in 0.5 mL methanol to formulate **L10**; 0.124 mg compound **6** in 0.5 mL methanol to formulate **L11**; 0.117 mg compound **7** in 0.5 mL methanol to formulate **L12**; 0.092 mg compound **8** in 0.5 mL methanol to formulate **L13**; 0.135 mg compound **9** in 0.5 mL methanol to formulate **L14** and **L15**), and LCP-1 (1 mg in 0.5 mL methanol) were mixed together in a 5 mL round bottom flask. All solvents were then very slowly removed using a rotary evaporator. A dry lipid film was produced on the flask walls with a glassy-clear appearance. The flask was kept under high vacuum (in a freeze dryer) overnight to remove the remaining solvent residues. The following day, the thin-film was rehydrated with 1 mL of Milli-Q water to produce multilamellar liposomes. CPP-free **L1** liposomes were prepared in the same manner. **L7** was prepared by simple mixing of **L1** with 0.067 mg of Tat_47–57_. Unilamellar liposomes (**L4** and **L15**) were produced using a mini extruder (Avantis Polar Lipids, Inc.) with 200 nm polycarbonate filters.

#### 2.2.4. Characterization of LCP-1 Loaded Liposomes

Liposome properties, including surface charge, particle size, size distribution, and polydispersity index (PDI), were characterized with dynamic light scattering (DLS). All measurements were performed at 25 °C with a back-scattering angle of 173°; eleven runs were performed per measurement and each measurement was repeated five times. The mean ± standard deviation was calculated based on the five measurements and the results were analyzed by Malvern Zetasizer software.

#### 2.2.5. *In-Vivo* Immunization

Outbred female Swiss (CD-1) mice (7–8 weeks old) obtained from the Animal Resource Centre (Perth, Western Australia) were used for the immunization study. The mice were housed in cages under sterile conditions and allowed to acclimatize for 7 days prior to experimentation. The mice were divided into experimental groups of five per group. All immunization protocols were approved by The University of Queensland Ethics Committee (Animal Ethics Unit, Office of Research Ethics, The University of Queensland; approval number: SCMB/AIBN/069/17) and conducted in compliance with the guidelines from the Australian National Health and Medical Research Council (NHMRC).

Immunization study 1**:**

On primary immunization (day 0), mice in the negative control group were intranasally administered with 30 μL (15 μL/nare) of PBS, while mice in the positive control group were intranasally immunized with P25-J8 (30 μg) and CTB (10 μg) dissolved in 30 μL (15 μL/nare) of endotoxin-free water. Mice in the seven test groups were given 30 μL (15 μL/nare) of freshly prepared **L1–L7** solution, equating to 30 µg of LCP-1 per mouse, respectively. Boosts (two total) were performed on days 14 and 28, with the same doses. Serum was collected via tail bleed on day −1, 13, and 27 and by cardiac puncture on day 38. The clear supernatant serum was collected after centrifugation for 10 min at 956 × *g* (3600 rpm). Serum samples were stored at −80 °C.

Immunization study 2:

Initially, intranasal immunizations were performed under anesthesia (isoflurane). However, the resulting IgG levels were highly inconsistent, even within groups, after three doses (see Supporting Information, Figure S14). Thus, a second immunization study was performed without anesthesia. On day 0, mice were intranasally immunized with freshly prepared **L1, L3** and **L8–L15** solutions at a dose of 30 μL (15 μL/nare) or PBS for the control group, as described above. Boosts were performed on days 21 and 42. Blood was collected via tail bleed on day −1, 20 and 41 and by cardiac puncture on day 52, and processed as detailed above to produce clear supernatant serum. Serum samples were stored at −80 °C.

#### 2.2.6. Determination of Antibody Titers

Enzyme-linked immunosorbent assays (ELISA) were used to determine the presence of J8-specific antibody (IgG, IgG1 and IgG2a) titers from the collected sera. J8 peptide (0.52 μg/well) was dissolved in 0.1 M sodium carbonate/bicarbonate (pH 9.6) coating buffer. Microtiter plates were coated with J8 peptide solution (100 µL/well) for 2 h at 37 °C, then blocked with 5% skim milk overnight at 4 °C to reduce non-specific binding. Serum samples were assessed based on serial two-fold dilutions, starting at a 1:100 dilution for serum IgG. Horseradish peroxidase-conjugated secondary antibodies (IgG, IgG1, and IgG2a) were added to the microtiter plates, followed by OPD substrate. The plates were incubated for 20 min in the dark at RT, then the optical density was measured at 450 nm. The antibody titers were described as the lowest possible dilution providing an absorbance of three standard deviations (SD) above the average absorbance of the control wells (serum from naive or PBS mice). Variation between the groups was assessed for statistical significance using one-way ANOVA followed by Tukey’s post hoc test with GraphPad Prism software.

#### 2.2.7. Indirect Bactericidal Assay

Opsonization assays were performed as previously reported [36,37]. Bacteria were streaked onto Todd Hewitt Broth (THB) agar plates supplemented with 5% yeast extract and incubated at 37 °C for 24 h. A single bacterial colony was transferred to THB (5 mL) supplemented with 5% yeast extract and incubated for another 24 h at 37 °C to obtain ~10^7^ colony forming units (CFU)/mL. Inactivated serum was heated in a water bath at 50 °C for 30 min. An aliquot (10 µL) of the diluted culture solution was mixed with heat-inactivated serum (10 μL) and horse blood (80 μL). The bacteria were incubated with the serum on a 96-well plate at 37 °C for 3 h. An aliquot (10 µL) from the culture material was analyzed based on CFUs counted from the plates. The plates were subsequently incubated at 37 °C for 24 h, and CFUs were counted. The opsonization activity of antibody serum (percent reduction in mean CFUs) was calculated (1 − [CFU in the presence of antipeptide sera]/[mean CFU in the presence of untreated media]) × 100). Opsonization assays were completed in duplicate.

#### 2.2.8. Cytotoxicity Study

The cytotoxicity of **L1**, **L3**, and **L14** was assessed using HEK-293 (human embryonic kidney) cell line, following previously reported methods [38,39]. HEK-293 Cells were seeded into 96-well microtiter assay plates at a density of 8 × 10^3^ cells in 190 μL per well of DMEM or RPMI complete media, respectively. The plates were incubated for 48 h at 37 °C and 5% CO_2_. **L1**, **L3**, and **L14** were tested over a series of four aliquots (10 μL) with concentrations of 1 mg/mL, 0.5 mg/mL, 0.25 mg/mL, and 0.125 mg/mL incubated for 72 h at 37 °C with 5% CO_2_. Positive control wells were treated with sodium dodecyl sulfate (SDS, 20% *w/v* in water), while negative control wells were treated with water (10 μL). Cell viability was measured by 3-(4,5-dimethylthiazol-2-yl)-2,5-diphenyltetrazolium bromide (MTT, 50 μg/well) assay. The absorbance of each well was measured at 570 nm, in duplicate.

## 3. Results

### 3.1. Preparation of CPP-Liposomes **L1**–**L15**

The previously-identified lead vaccine candidate LCP-1 [40], and CPP conjugates **1–9**, were synthesized using standard Boc-SPPS [41]. Conjugates **1–9** were designed to carry a lipidic group as a liposome anchoring moiety: two palmitic acids conjugated to the lysine moiety were used for this purpose. Variable lengths of spacers were placed between the lipidic moieties and Tat_47–57_ to identify more efficient CPP-liposome presentations. To synthesize compound **10** (see Supporting Information, Figure S12), DOPE-PEG_3400_-alkyne was initially produced according to the previously reported method [35]. Tat_47–57_ was synthesized by Boc-SPPS and further modified with 2-azidoacetic acid on its *N*-terminus to produce the product azide-Tat_47–57_. DOPE-PEG_3400_-alkyne and azide-Tat_47–57_ were conjugated via CuAAC reaction using copper wires [42] in DMF. Compounds **1–10** were further formulated into LCP-1-loaded liposomes to produce **L1–L15** (Table 1). **L1** was prepared using LCP-1, DPPC, and CH without any CPPs, while **L2–L15** were prepared using LCP-1, DPPC, CH, and CPP conjugates via thin-film hydration. After thin-film hydration, **L4** and **L15** were further extruded to produce unilamellar liposomes, while the other liposomes were maintained as multilamellar.

### 3.2. Characterization of CPP-Liposomes **L1**-**L15**

**L1**–**L15** were analyzed for particle size, PDI and zeta potential using DLS (Table 1). As expected, the diameter of multilamellar liposomes (**L1**–**L3**, **L5**–**L14**) varied greatly: particles size ranged from 70 nm to 5000 nm and the polydispersity was high (PDI 0.30–0.98). Zeta potentials ranged from 53 mV to 73 mV. The unilamellar liposome, **L4**, was produced as homogenous particles with a smaller diameter of 117 ± 1 nm, a lower PDI (0.14 ± 0.01) and a zeta potential of 37 ± 1 mV. Similarly, unilamellar liposome, **L15**, was also formed from uniform-sized particles with small diameter (112.4 ± 0.4 nm), narrow size distribution (PDI of 0.08 ± 0.01) and zeta potential 38 ± 2 mV.

### 3.3. Immunization

**Figure 2:**
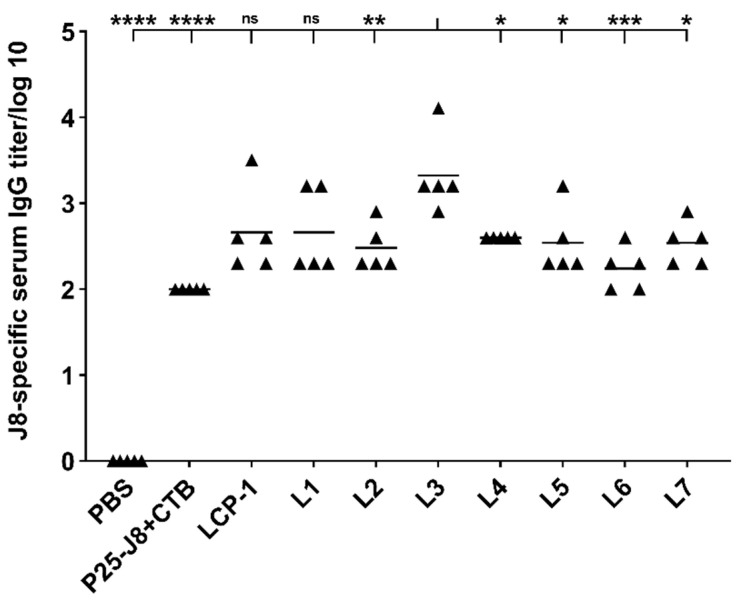
J8-specific antibody responses (log10) following intranasal administration of Tat_47–57_-based liposomes and controls in Swiss outbred mice (*n* = 5 per group), as determined by ELISA. Serum was collected at day 38 post-primary immunization. Statistical analysis was performed by one-way ANOVA followed by Tukey’s post hoc test to compare groups against **L3**, as indicated. Not significant (ns), *p* > 0.05; *, *p* < 0.05; **, *p* < 0.01; ***, *p* < 0.001; ****, *p* < 0.0001.

Tat_47–57_ is the most popular and intensively investigated CPP in vaccine delivery studies [10]. Thus, it was selected as a model CPP to optimize our CPP-liposome anchoring strategy. Outbred Swiss mice were intranasally administered with PBS, LCP-1 alone, CTB-adjuvanted antigen (P25-J8), or **L1–L7**. As a mucosal adjuvant, CTB was physically mixed with antigen P25-J8 to serve as the positive control, while PBS was used as the negative control. All groups received the same concentration of antigen (30 μg of LCP-1/mouse) in water (30 μL). The presence of J8-specific IgG antibodies in the serum was determined by ELISA. All liposomal formulations (**L1–L7**, Table 2), LCP-1, and the positive control (CTB+P25-J8) elicited significant J8-specific antibody titers in comparison to PBS in immunized mice (Figure 2). Mice administered with **L3**, which carried LCP-1 and compound **2** with a short spacer (Gly)_6_, triggered the highest antibody production among the tested liposomes. **L2** bearing **1 (**lipidated Tat_47–57_ without spacer) and **L6** bearing **10** (lipidated Tat_47–57_ with a long PEG_3400_ spacer) both produced significantly lower antibody titers than **L3**. Interestingly, when the concentration of the targeting moiety on the liposome’s surface was increased (**L5**), lower antibody titers were observed compared to those triggered by **L3**. Moreover, although multilamellar **L3** and unilamellar **L4** had the exact same composition, mice receiving **L3** produced significantly higher IgG titers than mice receiving unilamellar **L4.** No difference in IgG expression levels was detected among mice immunized with **L1** and **L7** (**L1** physically mixed with Tat_47–57_).

**Figure 3:**
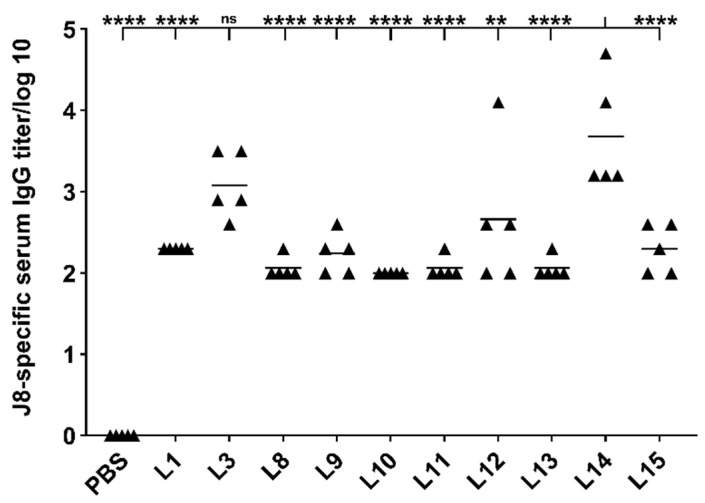
J8-specific antibody responses (log10) post-intranasal administration of CPP-based liposomes and controls in Swiss mice (*n* = 5 per group), as determined by ELISA from serum collected on day 52. Statistical analysis was performed by one-way ANOVA followed by Tukey’s post hoc test to compare groups against **L14**. Not significant (ns), *p* > 0.05; *, *p* < 0.05; **, *p* < 0.01; ***, *p* < 0.001; ****, *p* < 0.0001.

To determine which CPPs had the highest adjuvating potential, Swiss mice were intranasally immunized with CPP/LCP-1/liposome systems. The lead vaccine candidate from the anchoring strategy study (**L3**) was selected as a positive control. All liposome formulations tested produced higher serum IgG titers than mice immunized with the negative control (PBS) after three immunizations. Mice vaccinated with **L14**, bearing lipidated KALA, produced the highest J8-specific IgG titers in comparison to the other vaccinated groups (Figure 3). The **L14** antibody titers were higher even than **L3**; however, the difference was not statistically significant.

Small-sized unilamerllar liposomes (**L15**) induced a lower antibody response than their multilamellar equivalent, **L14**. Moreover, **L8** bearing Tat_47–57_ conjugated to palmitic acid via a longer linker (Gly)_12_ did not trigger stronger immune responses than **L3** bearing **2** with a short linker (Gly)_6_. Mice vaccinated with **L1**, **L8**, **L9**, **L10**, **L11**, **L13**, and **L15** elicited the same level of antibody production, which was significantly lower than that of **L14**.

**Figure 4:**
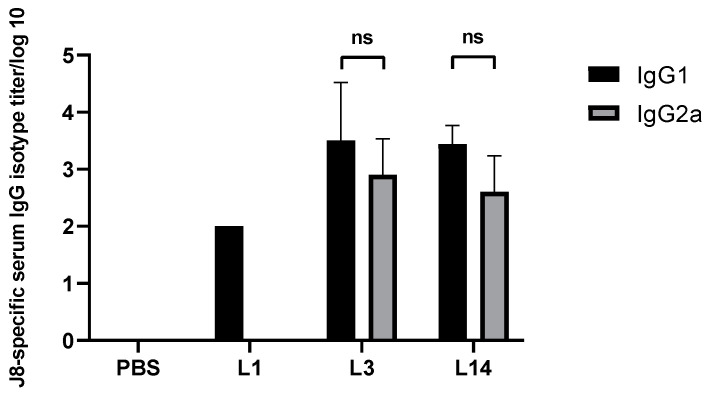
J8-specific IgG subclasses (log 10) at day 52 post primary immunization. Statistical analysis was performed using one-way ANOVA followed by Tukey’s post hoc test to compare with PBS-administered mice (ns, *p* > 0.05). Statistical analyses were performed using GraphPad Prism version 8.03.

In addition, IgG1 and IgG2a titers were measured for mice immunized with **L3** and **L14** by ELISA, and compared to **L1** (CPP free) and PBS (Figure 4). Similar IgG1/IgG2a levels were observed in **L3** and **L14** immunized mice, which correlates to balanced Th2/Th1 responses, respectively. In **L1** immunized mice only IgG1 were detected (Th2 responses)**.**

### 3.4. Evaluation of an Outbred Mouse Model for Opsonic Immune Responses Against GAS

**Figure 5:**
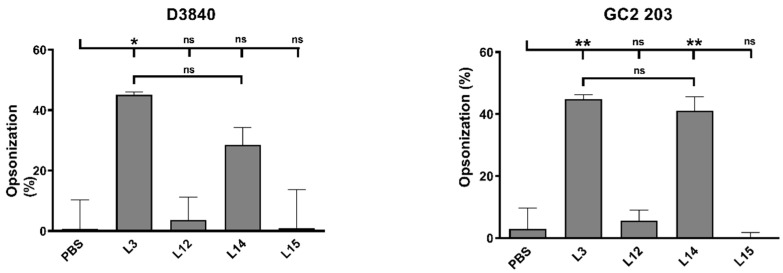
Average opsonization percentage of Group A Streptococcus strains (D3840 and GC2 203) by serum taken at day 52 after primary immunization in Swiss mice (*n* = 5 per group). Statistical analysis was performed using one-way ANOVA followed by Tukey’s post hoc test to compare with PBS-administered mice (ns, *p* > 0.05; * *p* < 0.05; ** *p* < 0.01;). Statistical analyses were performed using GraphPad Prism version 8.03.

To evaluate the quality of antibodies produced, an in vitro opsonization assay was performed against two clinically isolated GAS strains: D3840 and GC2 203. Five representative sera (PBS, **L3**, **L12**, **L14**, **L15**) were selected for the study. The sera obtained from mice vaccinated with **L3** and **L14** showed significantly higher opsonic activity against GC2 203 strains compared to the PBS group (Figure 5). Furthermore, serum from **L3**-vaccinated mice showed significant opsonic activity against D3840, and was slightly more effective than **L14** serum. Other tested sera were not opsonic.

## 4. Discussion

GAS pathogens infect humans primarily through the upper respiratory tract [28,43]. Thus, intranasal administration presents an attractive vaccine delivery route, as it mimics the natural route of the infection. Intranasal vaccines can also be self-administered and require no specialized equipment [44,45]. Nasal mucosal surfaces are highly vascularized, which could facilitate rapid antigen absorption into systemic circulation. Moreover, proteolytic enzyme activity associated with nasal mucosal tissue is low, significantly reducing the risk of antigen degradation. Importantly, the tissue primarily responsible for inducing immune responses following intranasal vaccine delivery is the nasal-associated lymphoid tissue (NALT). The similarities between mouse and human NALT substantially simplifies vaccine development and progression to clinical trials.

The use of peptide-based antigens is the most promising strategy for the development of GAS vaccines, as it eliminates the risk of inducing GAS-associated autoimmune responses [1]. Indeed, all GAS vaccines that have reached clinical trials in the past decades have been peptide-based. However, peptide vaccines require the use of strong, but safe, adjuvants. Here, we propose the use of CPPs as a self-adjuvanting moiety for GAS vaccines.

Over the past decade, CPPs have been investigated for antigen delivery to cells [10]. In vaccine constructs, CPPs are usually conjugated directly to the antigen. While this ensures co-delivery, it also increases the complexity of vaccine design. Most conjugations have been achieved through construction of a recombinant plasmid that expressed both antigen and CPP genes (for protein-based vaccines) [11,46,47,48,49,50], or a CPP/plasmid DNA complex (for DNA-based vaccines) [51,52]. On the contrary, here we designed a liposome-based delivery system that anchors both CPP and antigen/vaccine on the same liposomal nanoparticles to ensure co-delivery. To achieve this, the *N*-terminal of Tat_47–57_ and other CPPs were lipidated. This delivery system can be easily customized by: (a) introducing/modifying linkers between the lipid/s and CPPs; (b) modifying the anchoring lipids; and (c) regulating CPP concentration on the particle surface. Particle size and properties (e.g., through change of liposome lamellarity) are additionally controllable.

Previously, we demonstrated that LCP-1 bearing two C16 lipoamino acids (2-amino-d,l-hexadecanoic acid) can be effectively anchored into liposomes [53,54]. Therefore, CPPs were modified with two C16 fatty acids (palmitic acids) to achieve the same anchoring properties. To assess the necessity of such an anchoring strategy, a physical mixture of LCP-1/liposomes and Tat_47–57_ (**L7**) was prepared. The immune response induced by **L7** was similar to that of the CPP-free liposomes (**L1**: LPC-1/liposomes) and, as expected, was significantly lower than that of the CPP-anchored equivalent, **L3** (Figure 2). This suggests that lipidation of CPP is essential for the efficacy of liposome-based delivery systems. Additionally, lower IgG production was detected in mice treated with the positive control (physical mixture P25-J8/CTB) compared to mice receiving **L3,** suggesting an advantage of the CPP/liposome system over the standard adjuvating strategy.

The presentation of lipidated CPPs on the liposome surface can be regulated by the introduction of a spacer between the anchoring lipid moieties and the CPPs. Spacers with variable lengths were examined (see compounds **1**, **2**, **3**, **10**, Figure 1). Initially, simple lipidated Tat_47–57_ without spacer (**1**), with a short spacer (**2**), and with a long spacer (**10**) were examined. Among them, vaccine candidate **L3** bearing lipidated CPP **2** (with short linker) induced the highest IgG antibody titers against J8 (Figure 2). Extension of the polyGly spacer to 12 units (**3**) reduced the immunogenicity of the corresponding liposomes (**L8**) (Figure 3). Therefore, the presence of a short spacer (Gly)_6_ between the CPP and liposome was found to be optimal for vaccine efficacy. Similarly, Daudey and co-workers demonstrated that placing short spacers (equivalent of 4–8 glycines) between a model peptide, K_3_ (KIAALKE)_3_, and the anchoring lipid resulted in more effective interaction of K_3_ with biological membranes [55]; longer spacers were less effective.

It is well-known that CPP concentration has an impact on how these peptides and their cargo interact with the cell surface [56,57]. Thus, we explored two levels of CPP concentration in our vaccine delivery system. **L5** comprised a five-fold higher quantity of **2** compared to **L3** and all other liposomal formulations. Interestingly, mice treated with **L5** produced less IgG than mice treated with **L3** (Figure 2), which indicated that increasing CPP concentration might not necessary enhance CPP-mediated uptake of LCP-1-loaded liposomes. Low CPP concentration is generally regarded as preferable, as higher concentrations can be associated with adverse effect. For example, Transportan (GWTLNSAGYLLGKINLKALAALAKKIL) is toxic when its concentration exceeds 5 μM [58,59], and MAP (KLALKLALKALKAALKLA) has a strong toxic effect on various cell lines at concentrations over 1 μM [58,60]. Therefore, we also examined the potential toxicity of selected formulations (**L1**, **L3,** and **L14**); none were toxic to non-or cancerous human cell lines (see Supporting Information, Figure S14) and no noticeable side effects were observed in the vaccinated mice.

To further optimize our CPP-based vaccine delivery system, several additional CPPs were examined, including two other cationic CPPs: polyarginine and penetratin; three amphipathic CPPs: GV1001, KALA, and Pep-1, and one hydrophobic CPP: LAH4 (Figure 1). These CPPs were lipidated, incorporated with LCP-1-loaded liposomes (Figure 1) and examined together with **L1** and **L3** for their ability to trigger J8-specific IgG production in Swiss mice following intranasal immunization (Figure 3). **L1** and **L3** induced the same level of antibody titers as in the first immunization study (Figure 2). **L3** (bearing Tat_47–57_) and **L14** (bearing KALA) produced the highest IgG levels among the test liposomes (**L8**-**L15**. KALA-based liposomes were even more efficient than Tat_47–57_; however, the difference was not statistically significant. Interestingly, when the mice were intranasally immunized with the same vaccine candidates (**L1**, **L3**, **L8–L14**) under anesthesia using isoflurane, highly inconsistent IgG production levels were detected (see Supporting Information, Figure S15) and all liposomes stimulated significantly weaker immune responses. This may be explained by isoflurane’s ability to impair antigen uptake by APCs, which has been reported previously [61]. Moreover, anesthesia and body position can influence vaccine distribution in the nasal cavity and lungs [62,63,64].

Multilamellar liposomes have been reported to be as effective as small-sized unilamellar liposomes in inducing humoral immunity [53]. We tested both to identify which structure was more efficient in improving immune responses. Unilamellar **L4** bearing Tat_47–57_ produced a significantly lower IgG immune titer than multilamellar **L3** (Figure 2). To further confirm this observation, liposomes bearing KALA were also produced in two forms; again, multilamellar liposomes (**L14)** were more efficient in stimulating antibody production than unilamellar liposomes (**L15)** (Figure 3).

In our previous studies, LCP-1-based formulations predominantly produced IgG1 antibodies (Th2 response) [32,41,65]; however, a balanced Th1/Th2 response is preferable for vaccine efficacy and safety [66,67,68]. Here, mixed J8-specific Th1/Th2 immune responses were observed from the CPP-bearing formulations, **L3** and **L4**, while the CPP-free formulation (**L1**) provoked a Th2 (IgG1) response only. The balanced Th1/Th2 responses triggered by liposomal formulations **L3** and **L14** may be explained by the CPPs’ ability to enhance both cellular and humoral immune responses [10].

Induction of high antibody titers by a vaccine does not necessarily equate to high vaccine efficacy, as the antibodies produced may be inadequate at stopping bacterial growth. Therefore, an opsonization experiment was performed using serum from the mice immunized by the most effective formulations (**L3** and **L14**, bearing Tat_47–57_ and KALA, respectively), as well as selected low-efficacy liposomes (**L12** and unilamellar **L15**, bearing LAH4 and KALA, respectively) and PBS. Serum from mice treated with **L3** and **L14** induced high opsonic IgG antibody titers, while serum from the other three formulations failed to kill GAS at the tested concentration. This dramatic difference in opsonization capacity corresponds to the differences in antibody response; for example, **L14** induced 15-fold higher antibody titers than **L15** (Figure 3). Given these findings, both Tat_47–57_- and KALA-based liposomes are promising delivery systems for intranasally administered lipopeptide-based vaccines.

## 5. Conclusions

We demonstrated that the incorporation of Tat_47–57_ in an LCP-1-based liposomal delivery system enhanced J8-specific antibody production when: (a) the CPPs were anchored to liposomes; (b) an appropriate spacer was used between CPPs and the lipidic anchoring moieties; (c) CPPs were presented in relatively low concertation; and (d) multilamellar liposomes were employed. Moreover, the vaccine candidate bearing Tat_47–57_ produced a significantly higher immune response compared with a physical mixture of peptide antigen and the commercial adjuvant, CTB, which suggested that lipidated Tat_45–57_ could be considered as a potential alternative to classic adjuvants. We also compared a variety of CPPs for their immunostimulating/delivery potency for the first time. We found that of those tested, only Tat_47–57_ and KALA, upon incorporation into liposomes, induced the production of high levels of opsonic antibodies against GAS. In addition, Tat_47–57_ and KALA-based formulations induced well-balanced Th1/Th2 responses, desirable for vaccine efficacy and safety. In summary, CPPs have the potential to improve humoral immune responses and, in combination with liposomes, provide more potent immune stimulation than the classical adjuvant CTB.

## Figures and Tables

**Table 1:** Composition and physicochemical characterization of LCP-1-loaded liposomes (**L1–L15**).

LCP-1-Loaded Liposomes	CPP Conjugates	Liposome Classification	Particle Size Range (d.nm)	Polydispersity Index (PDI)	Zeta Potential (mV)
**L1**	**-**	M	100–5000	0.30 ± 0.20	60 ± 1
**L2**	**1**	M	100–1400	0.98 ± 0.02	72 ± 1
**L3**	**2**	M	80–2300	0.63 ± 0.03	67 ± 2
**L4**	**2**	U	117 ± 1	0.14 ± 0.01	37 ± 1
**L5**	**2 ^a^**	M	200–3000	0.81 ± 0.02	73 ± 1
**L6**	**10**	M	200–5000	0.65 ± 0.13	57 ± 1
**L7**	**Tat_47–57_^b^**	M	100–5000	0.82 ± 0.04	68 ± 1
**L8**	**3**	M	250–5000	0.47 ± 0.11	62 ± 2
**L9**	**4**	M	100–5000	0.88 ± 0.03	64 ± 2
**L10**	**5**	M	300–5000	0.48 ± 0.04	62 ± 0
**L11**	**6**	M	100–5000	0.74 ± 0.08	55 ± 3
**L12**	**7**	M	70–2000	0.10 ± 0.01	53 ± 2
**L13**	**8**	M	100–3000	0.58 ± 0.13	64 ± 2
**L14**	**9**	M	100–4000	0.62 ± 0.07	57 ± 3
**L15**	**9**	U	112.4 ± 0.4	0.08 ± 0.01	38 ± 2

**^a^** A five-fold higher concentration of **2** was loaded into the liposome formulation; **^b^** Physical mixture of Tat_47–57_ and LCP-1-loaded liposomes; M-multilamellar; U-unilamellar.

**Table 2:** Composition of CPP conjugates comprised in LCP-1 loaded liposomes (**L1**–**L15**).

LCP-1-Loaded Liposomes	CPP Conjugate Composition			Liposome Classification
	CPP	Lipid Moiety	Spacer	
**L1**	-	-	-	M
**L2**	Tat_47–57_	(Palmitic acid)_2_	-	M
**L3**	Tat_47–57_	(Palmitic acid)_2_	(Gly)_6_	M
**L4**	Tat_47–57_	(Palmitic acid)_2_	(Gly)_6_	U
**L5**	Tat_47–57_^a^	(Palmitic acid)_2_	(Gly)_6_	M
**L6**	Tat_47–57_	DOPE	PEG_3400_	M
**L7**	Tat_47–57_^b^	-	-	M
**L8**	Tat_47–57_	(Palmitic acid)_2_	(Gly)_12_	M
**L9**	Polyarginine	(Palmitic acid)_2_	(Gly)_6_	M
**L10**	Penetratin	(Palmitic acid)_2_	(Gly)_6_	M
**L11**	Pep-1	(Palmitic acid)_2_	(Gly)_6_	M
**L12**	LAH4	(Palmitic acid)_2_	(Gly)_6_	M
**L13**	GV1001	(Palmitic acid)_2_	(Gly)_6_	M
**L14**	KALA	(Palmitic acid)_2_	(Gly)_6_	M
**L15**	KALA	(Palmitic acid)_2_	(Gly)_6_	U

**^a^** A five-fold higher concentration of Tat _47–57_ was loaded into the liposome formulation; **^b^** Physical mixture of Tat_47–57_ and LCP-1 loaded liposomes; M, multilamellar; U, unilamellar.

## Data Availability

The data presented in this study are available in article and Supplementary Materials.

## Supplementary Materials

The following are available online at https://www.mdpi.com/article/10.3390/vaccines9050499/s1, Figure S1: ESI-MS spectrum and analytical RP-HPLC spectrum of LCP-1, Figure S2: ESI-MS spectrum and analytical RP-HPLC spectrum of Tat_47–57_, Figure S3: ESI-MS spectrum and analytical RP-HPLC spectrum of compound **1**, Figure S4: ESI-MS spectrum and analytical RP-HPLC spectrum of compound **2**, Figure S5: ESI-MS spectrum and analytical RP-HPLC spectrum of compound **3**, Figure S6: ESI-MS spectrum and analytical RP-HPLC spectrum of compound **4**, Figure S7: ESI-MS spectrum and analytical RP-HPLC spectrum of compound **5**, Figure S8: ESI-MS spectrum and analytical RP-HPLC spectrum of compound **6**, Figure S9: ESI-MS spectrum and analytical RP-HPLC spectrum of compound **7**, Figure S10: ESI-MS spectrum and analytical RP-HPLC spectrum of compound **8**, Figure S11: ESI-MS spectrum and analytical RP-HPLC spectrum of compound **9**, Figure S12: The click chemistry of compound **10**. Compound **10** was produced based on a copper-catalyzed alkyne-azide 1,3-dipolar cycloaddition (CuAAC) ‘‘click” reaction to conjugate DOPE-PEG_3400_-alkyne to the azide-Tat_47–57_, Figure S13: The MALDI-TOF Mass Spectrometry of DOPE-PEG_3400_-alkyne (molecular weight Range: 4000–4200) (a) and DOPE-PEG_3400_-Tat_47–57_ (molecular weight Range: 5558–5758) (compound **10**), Figure S14: The cytotoxicity results for **L1**, **L3** and **L14** against HEK-293: human kidney cell line. Values are reported as mean: standard error of the mean (SEM), Figure S15: J8-specific antibody responses (log10) post-intranasal administration of CPP-based liposomes and controls in Swiss mice (*n* = 5), as determined by ELISA. The immunization study was performed with the external isoflurane for all intranasal immunization procedure. Serum collected at day 52, 10 days after three boosters on day 14 and 28, 42. Statistical analysis was performed by one-way ANOVA followed by Tukey’s post hoc test to compare test groups against PBS. Not significant (ns), *p* > 0.05; *, *p* < 0.05; **, *p* < 0.01; ***, *p* < 0.001; ****, *p* < 0.0001).
