# Supplementary material for: Cell-Penetrating Peptides-Based Liposomal Delivery System Enhanced Immunogenicity of Peptide-Based Vaccine against Group A Streptococcus
Source: Vaccines (Basel). 2021 May 12;9(5):499. doi: 10.3390/vaccines9050499 (PMC8151947; doi:10.3390/vaccines9050499)
Supplement: Supplementary file 1 [file vaccines-09-00499-s001.zip › vaccines-1172591-supplementary.pdf]

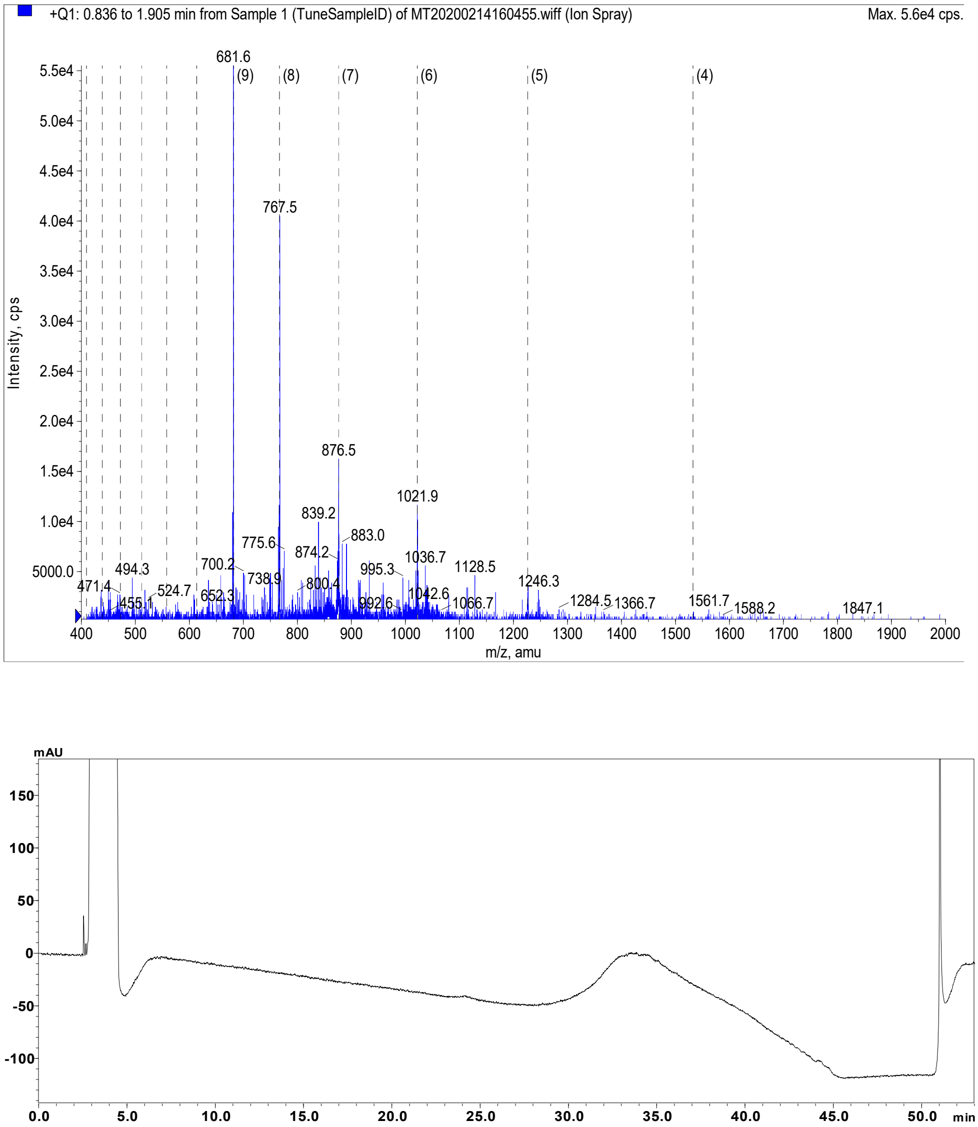

Figure S1. ESI-MS spectrum and analytical RP-HPLC spectrum of LCP-1.

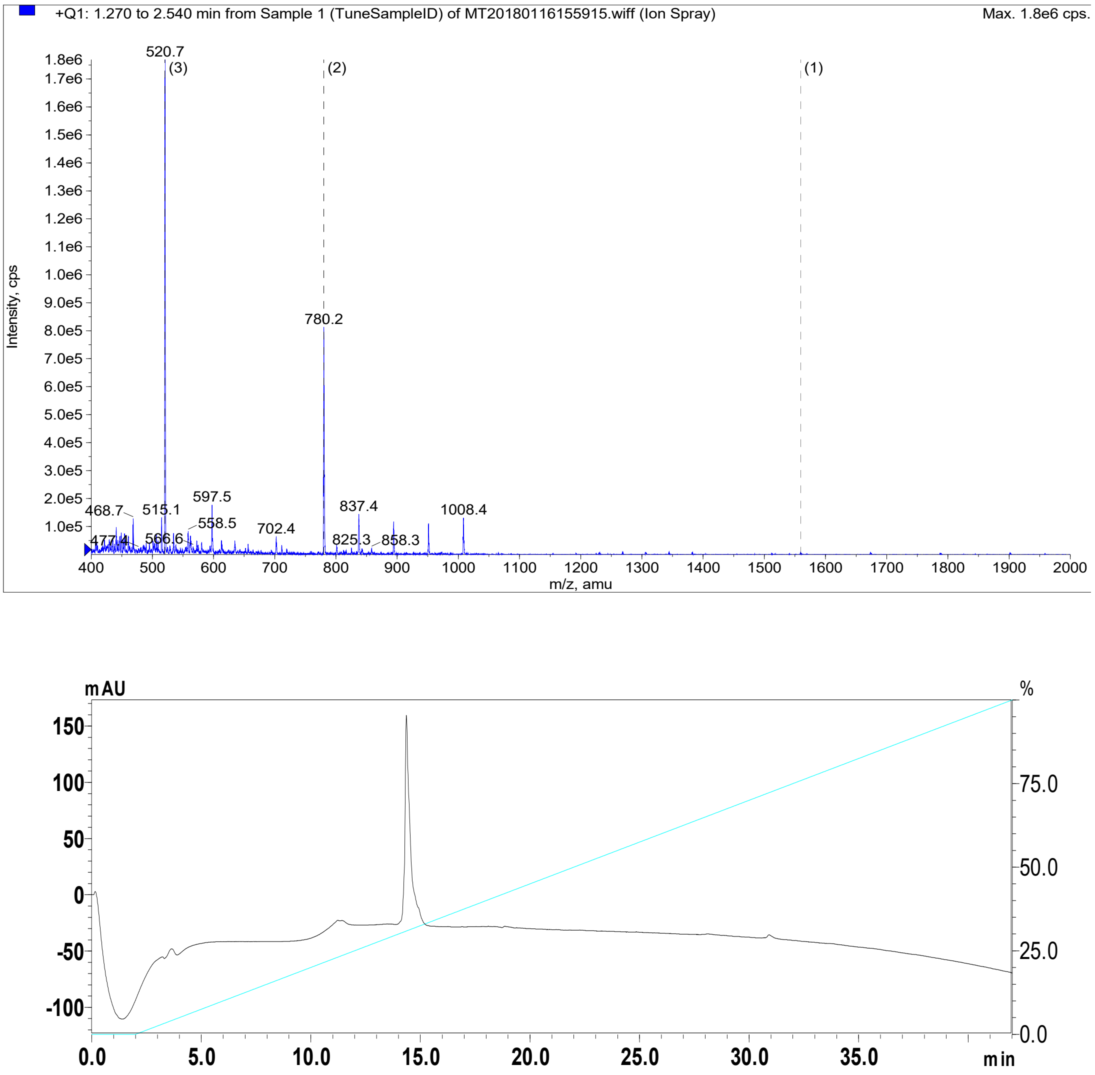

Figure S2. ESI-MS spectrum and analytical RP-HPLC spectrum of Tat$_{47-57}$.

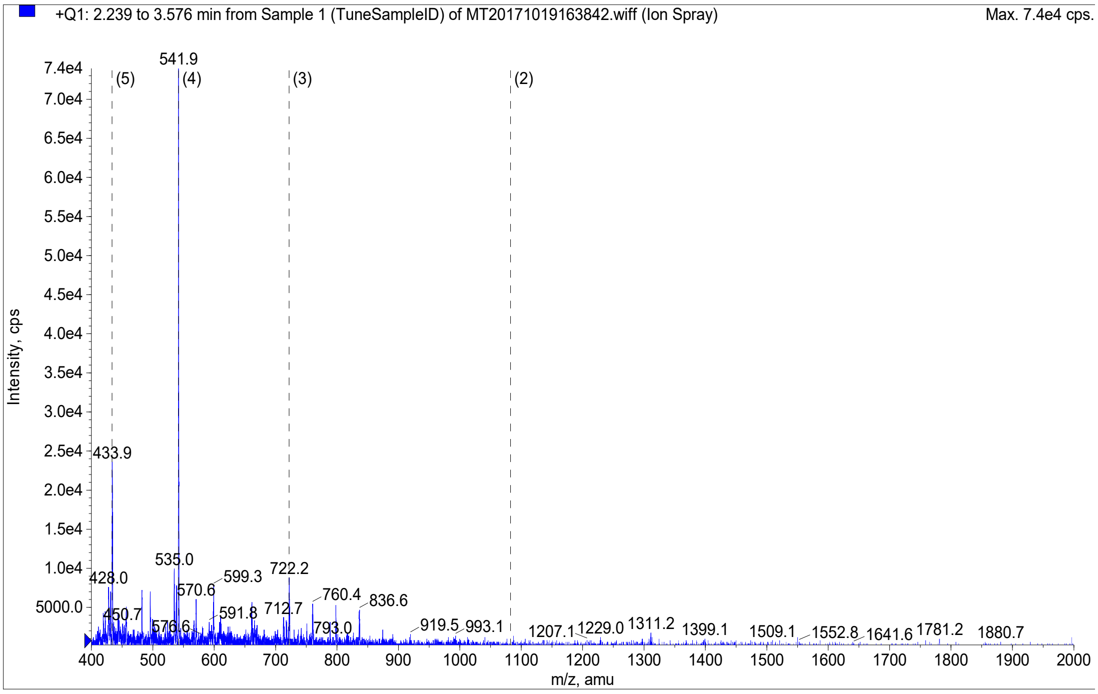

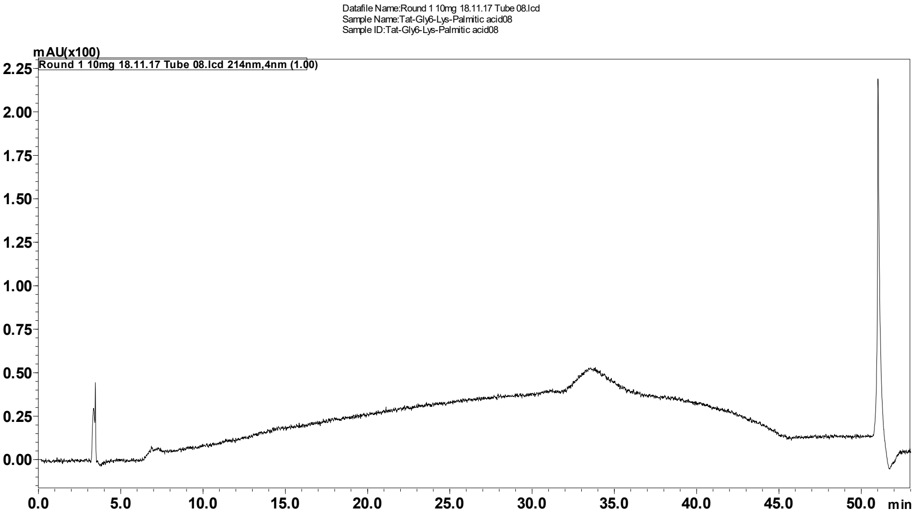

Figure S3. ESI-MS spectrum and analytical RP-HPLC spectrum of compound **1**.

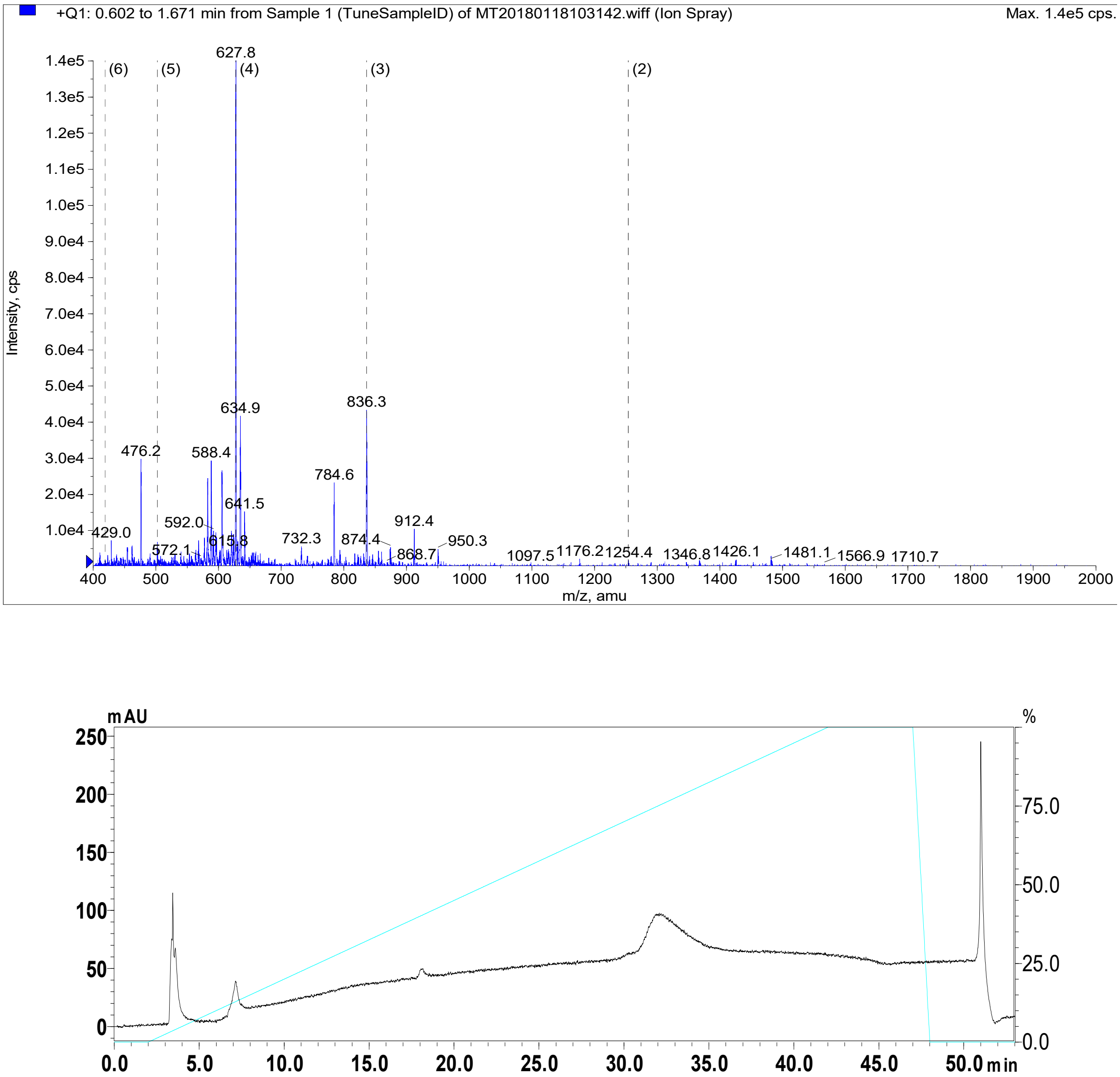

Figure S4. ESI-MS spectrum and analytical RP-HPLC spectrum of compound **2**.

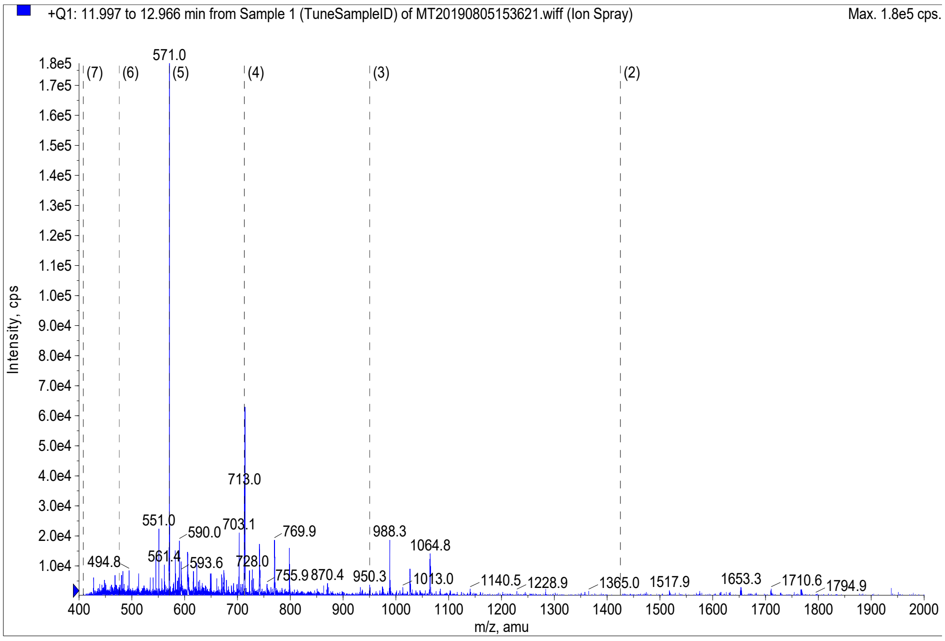

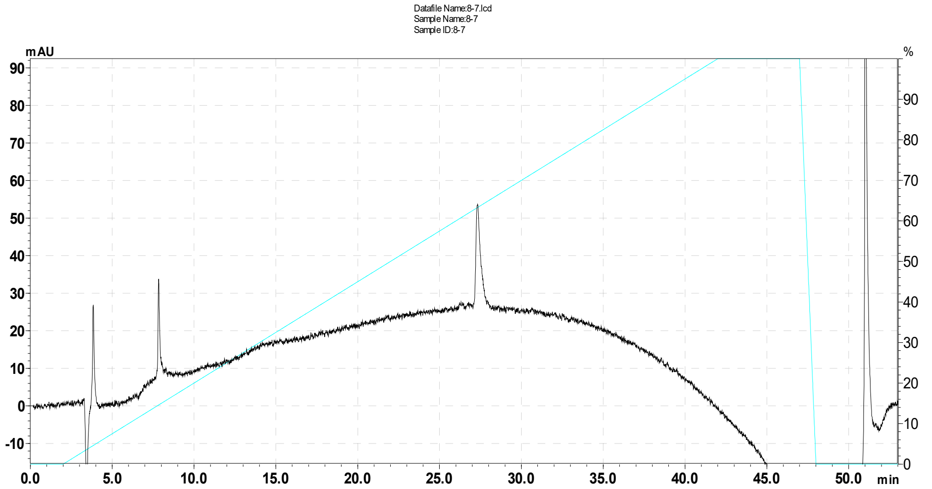

Figure S5. ESI-MS spectrum and analytical RP-HPLC spectrum of compound **3**.

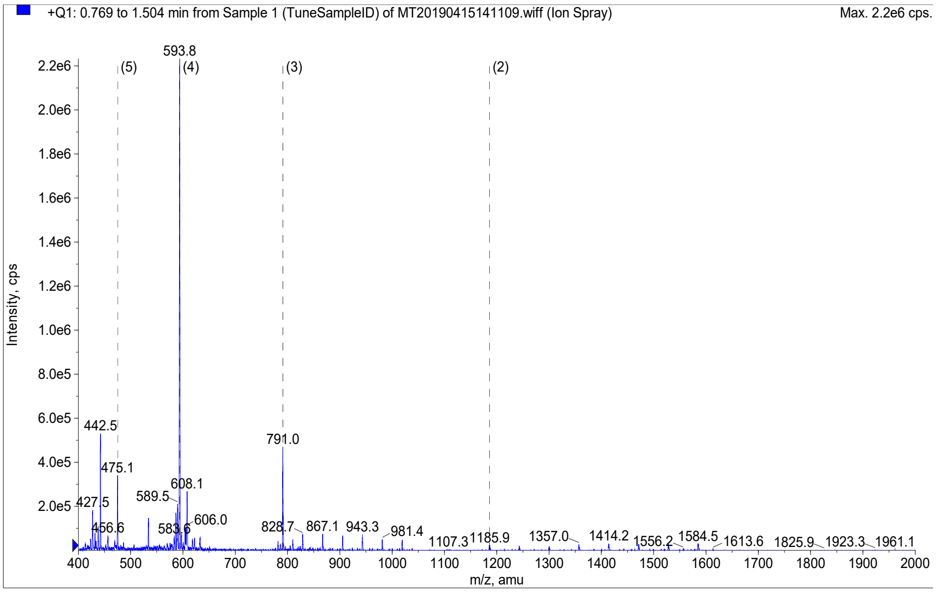

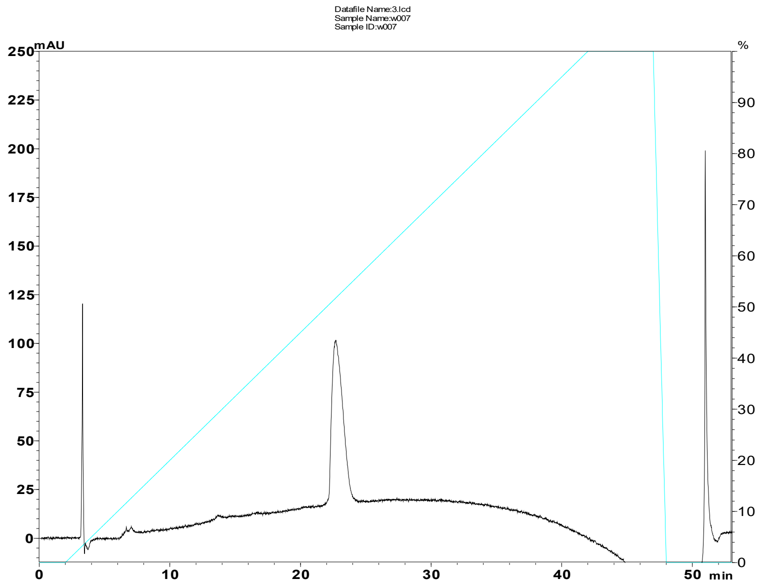

Figure S6. ESI-MS spectrum and analytical RP-HPLC spectrum of compound **4**.

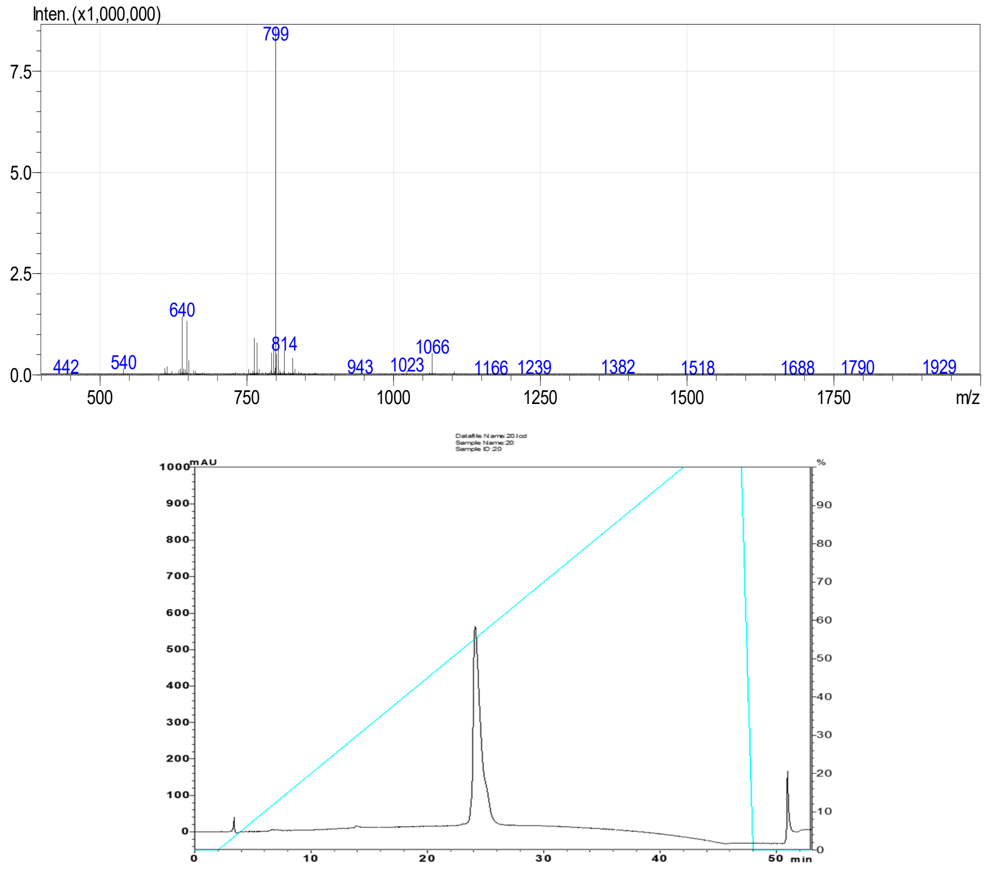

Figure S7. ESI-MS spectrum and analytical RP-HPLC spectrum of compound **5**.

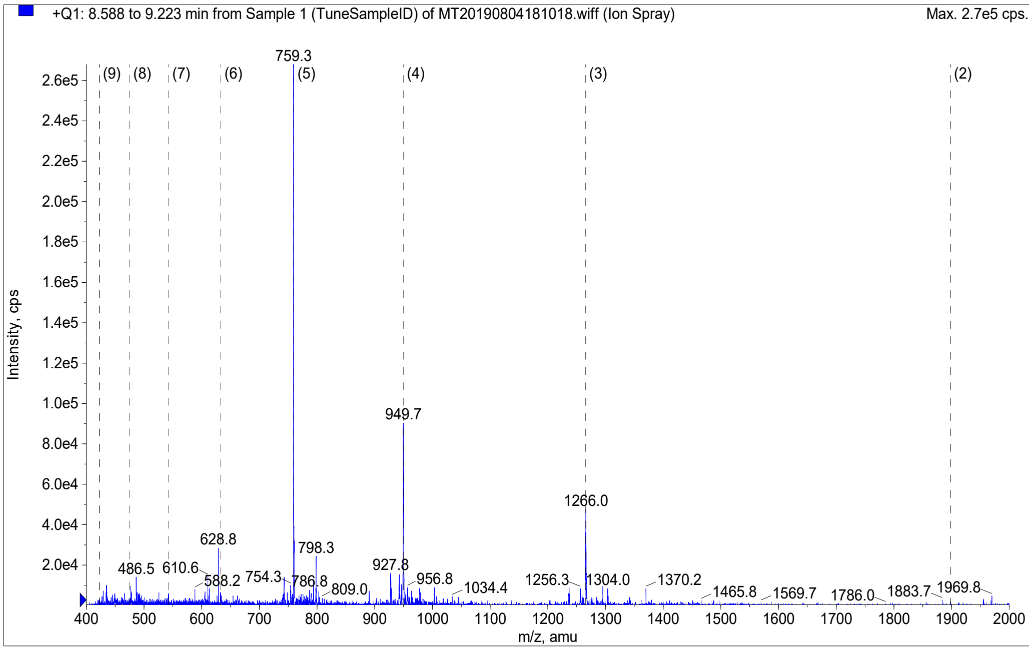

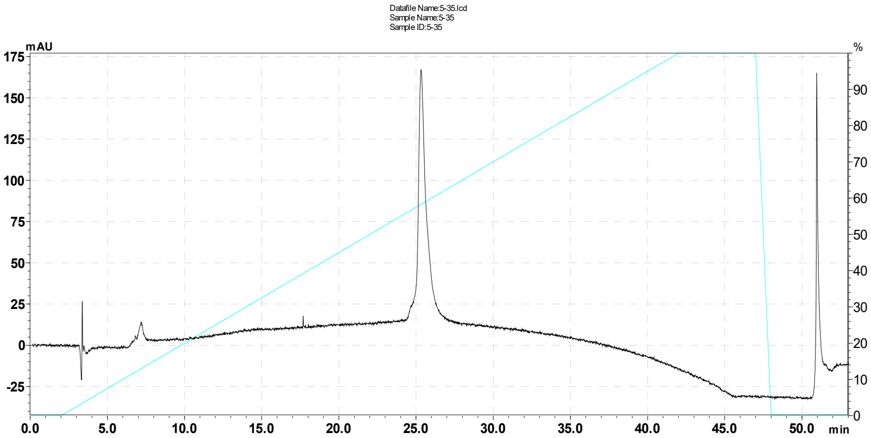

Figure S8. ESI-MS spectrum and analytical RP-HPLC spectrum of compound **6**.

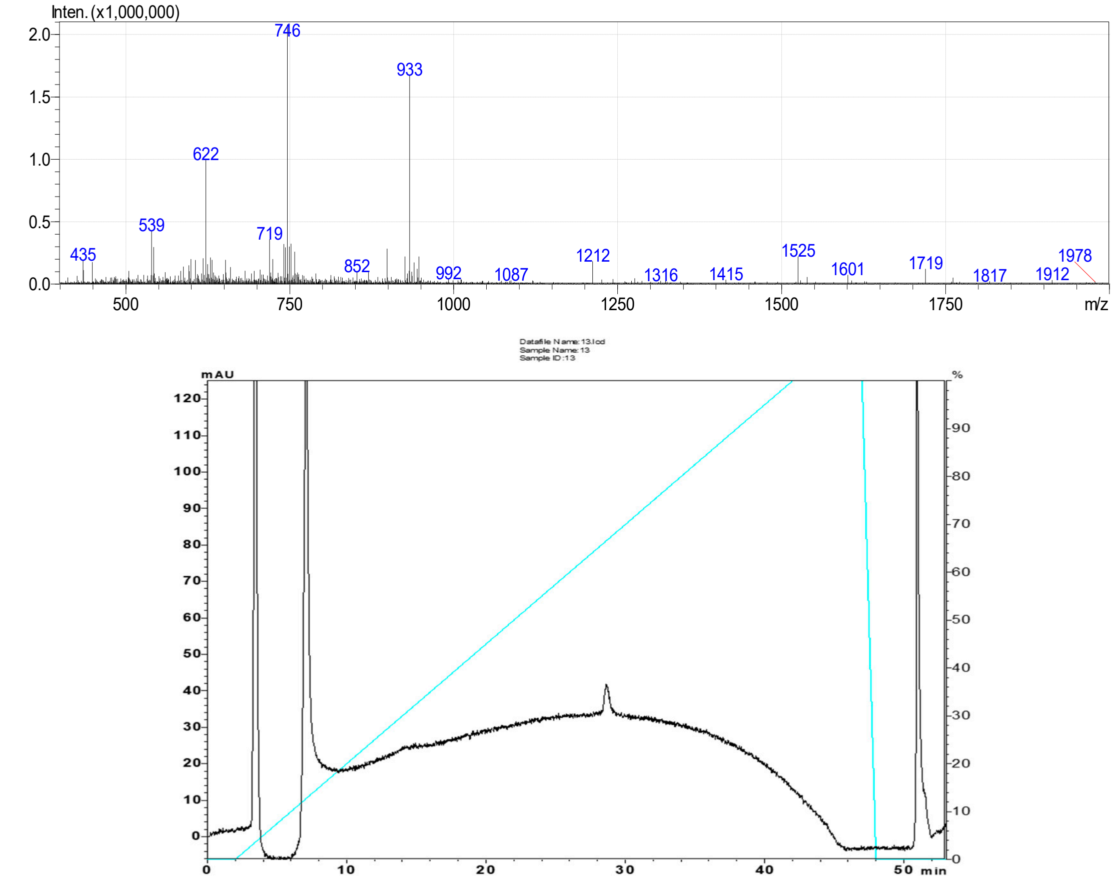

Figure S9. ESI-MS spectrum and analytical RP-HPLC spectrum of compound **7**.

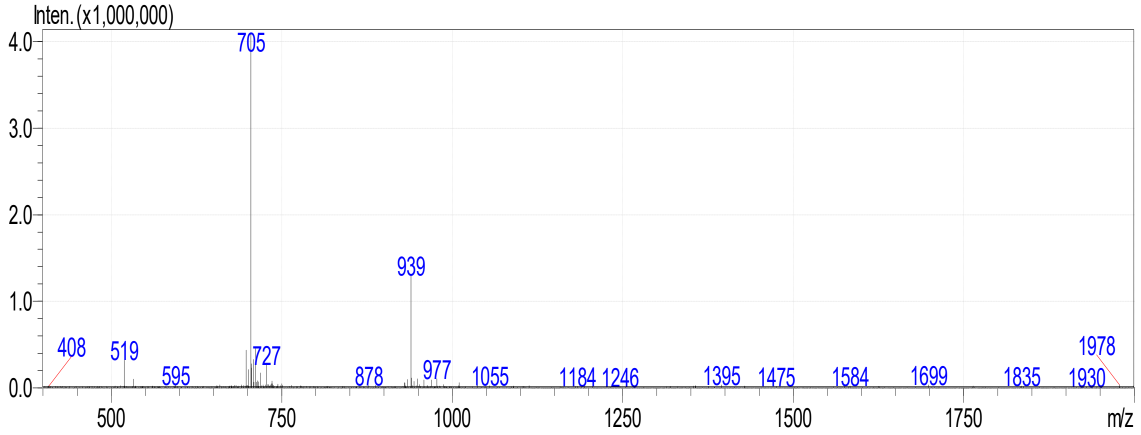

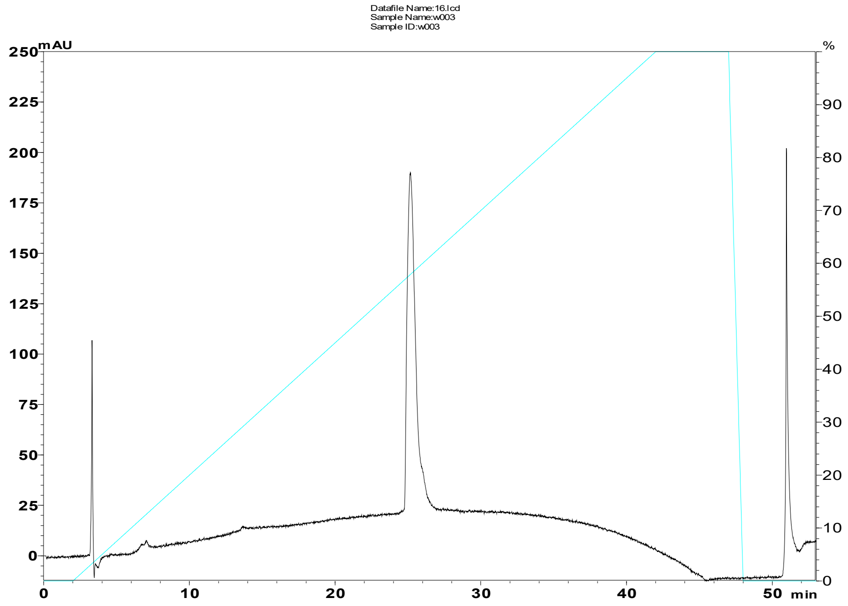

Figure S10. ESI-MS spectrum and analytical RP-HPLC spectrum of compound **8.**

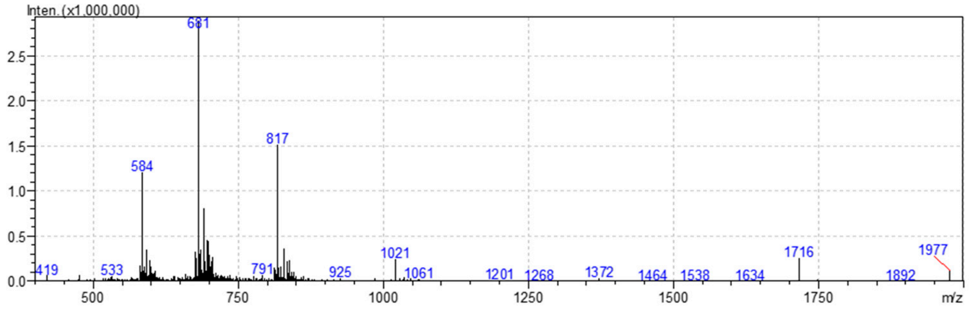

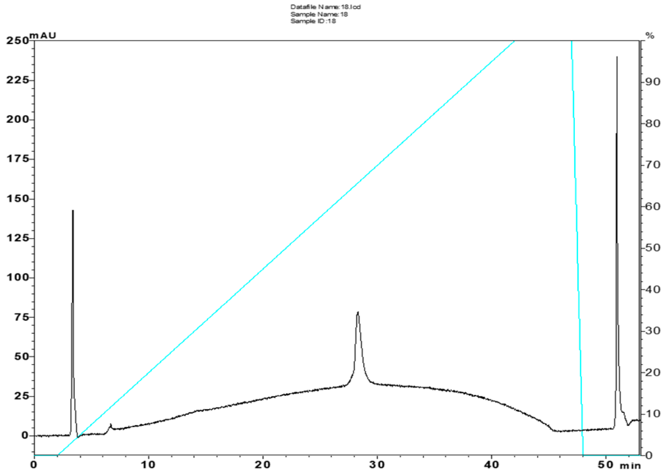

Figure S11. ESI-MS spectrum and analytical RP-HPLC spectrum of compound **9**.

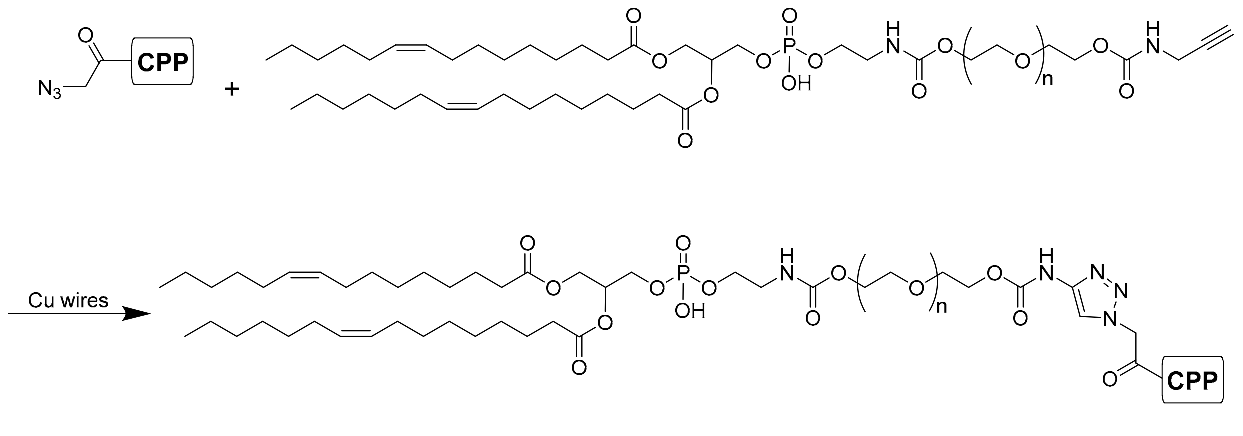

10: n ≈ 77, CPP = Tat_{47-57}

Figure S12. The click chemistry of compound **10**. Compound **10** was produced based on a copper-catalyzed alkyne-azide 1,3-dipolar cycloaddition (CuAAC) ''click'' reaction to conjugate DOPE-PEG_{3400}-alkyne to the azide-Tat_{47-57}.

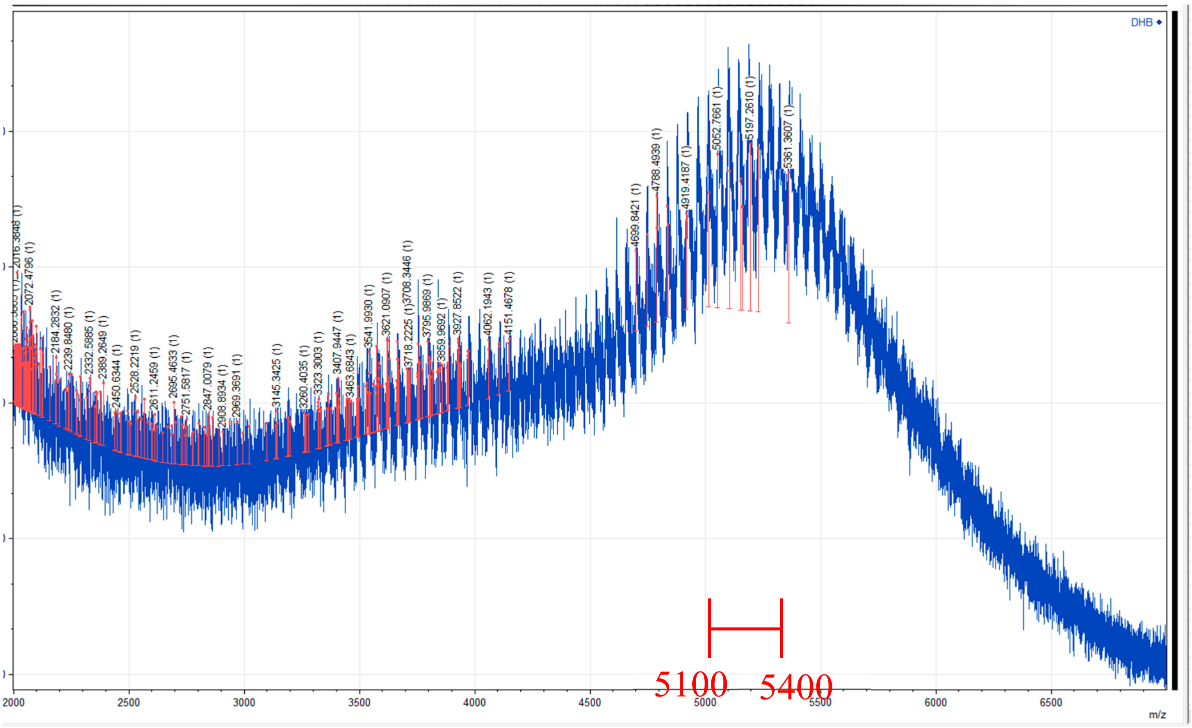

Figure S13. The MALDI-TOF Mass Spectrometry of DOPE-PEG$_{3400}$-Tat$_{47-57}$ (molecular weight Range: 5558-5758) (compound **10**)

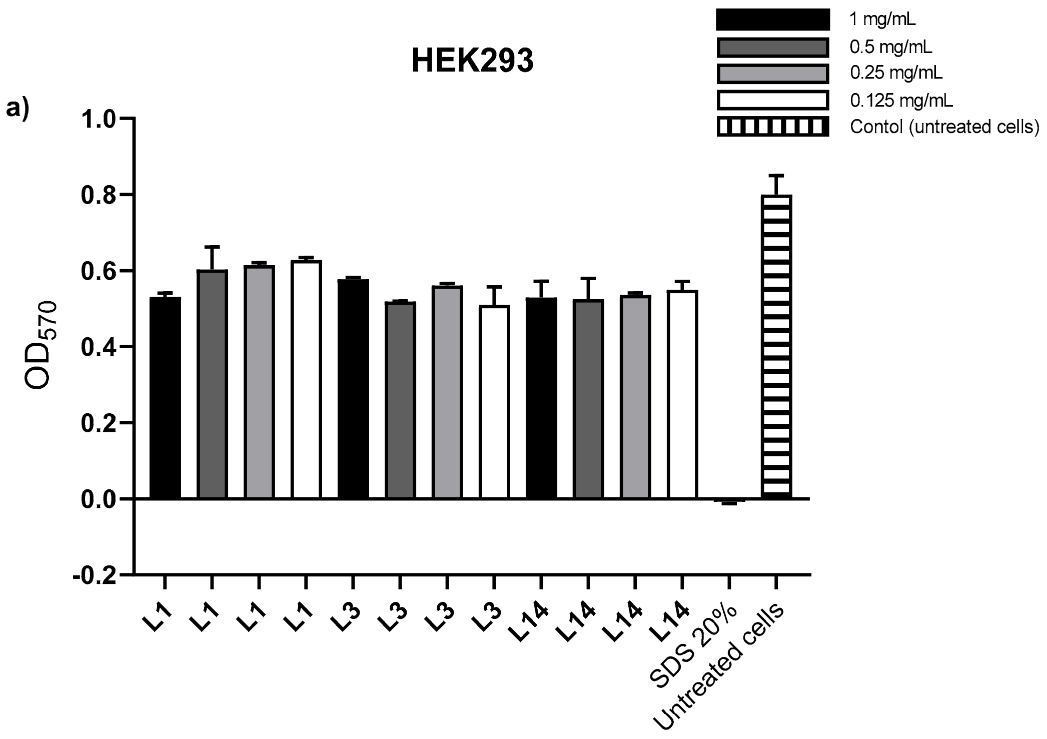

Figure S14. The cytotoxicity results for **L1**, **L3** and **L14** against HEK-293: human kidney cell line. Values are reported as mean: standard error of the mean (SEM).

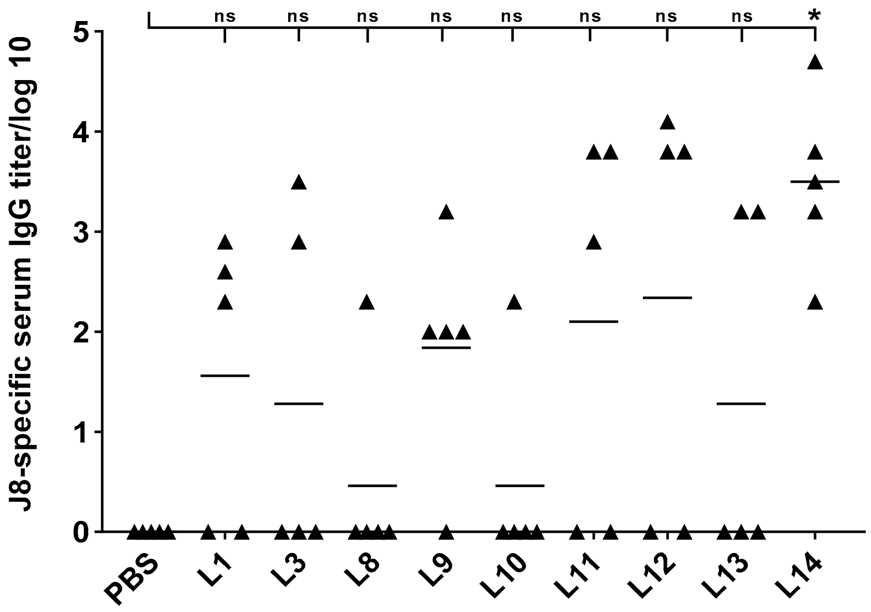

Figure S15. J8-specific antibody responses (log10) post-intranasal administration of CPP-based liposomes and controls in Swiss mice (n = 5), as determined by ELISA. The immunization study was performed with the external isoflurane for all intranasal immunization procedure. Serum collected at day 52, 10 days after three boosters on day 14 and 28, 42. Statistical analysis was performed by one-way ANOVA followed by Tukey's post hoc test to compare test groups against PBS. Not significant (ns), $p > 0.05$; *, $p < 0.05$; **, $p < 0.01$; ***, $p < 0.001$; ****, $p < 0.0001$.
